# Supplementary material for: Predictors of COVID-19 vaccination intention among students in Ghana: An application of the Health Belief Model and Theory of Planned Behaviour
Source: PLOS Glob Public Health. 2025 Dec 29;5(12):e0005561. doi: 10.1371/journal.pgph.0005561 (PMC12747339; doi:10.1371/journal.pgph.0005561)
Supplement: S1 Table — (DOCX) [file pgph.0005561.s002.docx]

**S1 Table. Univariate analysis of COVID-19 vaccination intention by sociodemographic, health-related characteristics, and adherence to preventive protocols**

| **Variables** | **Intention to vaccinate against COVID-19** | | | | **Total = 420 (100%)** | | **Chi-square (**$\boldsymbol{x}^{\boldsymbol{2}}$**)** | **p-value** |
| --- | --- | --- | --- | --- | --- | --- | --- | --- |
|  | **No = 163 (38.8%)** | | **Yes = 257 (61.2%)** | |  |  |  |  |
|  | **n (%)** | | **n (%)** | | **n (%)** | |  |  |
| **Demographics** |  | |  | |  | |  |  |
| **Age mean ± std dev.** | **17.89 ± 1.62** | | **18.35 ± 1.89** | | **18.21 ± 1.79** | |  |  |
| **Age group (years)** |  |  |  |  |  |  | 2.38 | 0.123 |
| 15-17 | 62 | (38.0) | 79 | (30.7) | 141 | (33.6) |  |  |
| 18-30 | 101 | (62.0) | 178 | (69.3) | 279 | (66.4) |  |  |
| **Sex** |  |  |  |  |  |  | 3.97 | **0.046*** |
| Female | 70 | (42.9) | 136 | (52.9) | 206 | (49.0) |  |  |
| Male | 93 | (57.1) | 121 | (47.1) | 214 | (51.0) |  |  |
| **Religious affiliation** |  |  |  |  |  |  | 2.74 | 0.255 |
| Christianity | 144 | (88.3) | 220 | (85.6) | 364 | (86.7) |  |  |
| Islam | 19 | (11.7) | 33 | (12.8) | 52 | (12.4) |  |  |
| African Traditional Religion | 0 | (0.0) | 4 | (1.6) | 4 | (1.0) |  |  |
| **Ethnic group** |  |  |  |  |  |  | 6.22 | 0.399 |
| Akan | 15 | (9.2) | 36 | (14.0) | 51 | (12.1) |  |  |
| Buem | 32 | (19.6) | 42 | (16.3) | 74 | (17.6) |  |  |
| Ewe | 71 | (43.6) | 92 | (35.8) | 163 | (38.8) |  |  |
| Guan | 6 | (3.7) | 13 | (5.1) | 19 | (4.5) |  |  |
| Hausa | 16 | (9.8) | 24 | (9.3) | 40 | (9.5) |  |  |
| Konkomba | 16 | (9.8) | 33 | (12.8) | 49 | (11.7) |  |  |
| Others | 7 | (4.3) | 17 | (6.6) | 24 | (5.7) |  |  |
| **School attended** |  |  |  |  |  |  | 4.89 | 0.18 |
| Baglo Ridge SHS | 33 | (20.2) | 57 | (22.2) | 90 | (21.4) |  |  |
| Bueman SHS | 51 | (31.3) | 57 | (22.2) | 108 | (25.7) |  |  |
| Father Dogli SHS | 39 | (23.9) | 78 | (30.4) | 117 | (27.9) |  |  |
| Okadjakrom SHTS | 40 | (24.5) | 65 | (25.3) | 105 | (25.0) |  |  |
| **Academic level** |  |  |  |  |  |  | 2.49 | 0.114 |
| SHS 2 (Second year) | 104 | (63.8) | 144 | (56.0) | 248 | (59.0) |  |  |
| SHS 3 (Third year) | 59 | (36.2) | 113 | (44.0) | 172 | (41.0) |  |  |
| **Course of study** |  |  |  |  |  |  | 18.01 | **0.012*** |
| Business | 8 | (4.9) | 5 | (1.9) | 13 | (3.1) |  |  |
| Fashion Design | 5 | (3.1) | 22 | (8.6) | 27 | (6.4) |  |  |
| General Agriculture | 2 | (1.2) | 6 | (2.3) | 8 | (1.9) |  |  |
| General Arts | 64 | (39.3) | 84 | (32.7) | 148 | (35.2) |  |  |
| General Science | 7 | (4.3) | 12 | (4.7) | 19 | (4.5) |  |  |
| Home Economics | 14 | (8.6) | 46 | (17.9) | 60 | (14.3) |  |  |
| Technical | 57 | (35.0) | 78 | (30.4) | 135 | (32.1) |  |  |
| Visual Art | 6 | (3.7) | 4 | (1.6) | 10 | (2.4) |  |  |
| **Residency status** |  |  |  |  |  |  | 0.45 | 0.504 |
| Resident | 139 | (85.3) | 225 | (87.5) | 364 | (86.7) |  |  |
| Non-resident | 24 | (14.7) | 32 | (12.5) | 56 | (13.3) |  |  |
| **Health related variables** |  |  |  |  |  |  |  |  |
| **History of COVID-19** |  |  |  |  |  |  | 7.25 | **0.007*** |
| No | 158 | (96.9) | 231 | (89.9) | 389 | (92.6) |  |  |
| Yes | 5 | (3.1) | 26 | (10.1) | 31 | (7.4) |  |  |
| **family history of underlying conditions** |  |  |  |  |  |  | 1.74 | 0.187 |
| No | 151 | (92.6) | 228 | (88.7) | 379 | (90.2) |  |  |
| Yes | 12 | (7.4) | 29 | (11.3) | 41 | (9.8) |  |  |
| **Perception of good health** |  |  |  |  |  |  | 0.01 | 0.916 |
| No | 43 | (26.4) | 69 | (26.8) | 112 | (26.7) |  |  |
| Yes | 120 | (73.6) | 188 | (73.2) | 308 | (73.3) |  |  |
| **Adherence to COVID-19 protocol** |  |  |  |  |  |  |  |  |
| **Face mask usage** |  |  |  |  |  |  | 0.13 | 0.716 |
| No | 58 | (35.6) | 87 | (33.9) | 145 | (34.5) |  |  |
| Yes | 105 | (64.4) | 170 | (66.1) | 275 | (65.5) |  |  |
| **Handwashing practices** |  |  |  |  |  |  | 5.40 | **0.02*** |
| No | 20 | (12.3) | 15 | (5.8) | 35 | (8.3) |  |  |
| Yes | 143 | (87.7) | 242 | (94.2) | 385 | (91.7) |  |  |
| **Social distancing practices** |  |  |  |  |  |  | 10.15 | **0.001*** |
| No | 82 | (50.3) | 89 | (34.6) | 171 | (40.7) |  |  |
| Yes | 81 | (49.7) | 168 | (65.4) | 249 | (59.3) |  |  |
| **Hand sanitiser use** |  |  |  |  |  |  | 0.31 | 0.581 |
| No | 36 | (22.1) | 51 | (19.8) | 87 | (20.7) |  |  |
| Yes | 127 | (77.9) | 206 | (80.2) | 333 | (79.3) |  |  |
| *p-value less than 0.05 | | | | | | | | |
