# Supplementary material for: Predictors of COVID-19 vaccination intention among students in Ghana: An application of the Health Belief Model and Theory of Planned Behaviour
Source: PLOS Glob Public Health. 2025 Dec 29;5(12):e0005561. doi: 10.1371/journal.pgph.0005561 (PMC12747339; doi:10.1371/journal.pgph.0005561)
Supplement: S2 Table — (DOCX) [file pgph.0005561.s003.docx]

**S2 Table. Model fit statistics for hierarchical logistic regression models predicting COVID-19 vaccination intention**

| **Statistic** | **Model 1** | **Model 2** | **Model 3** |
| --- | --- | --- | --- |
| Log-likelihood Model | -248.799 | -247.420 | -236.387 |
| Log-likelihood Intercept-only | -280.513 | -280.513 | -280.513 |
| Deviance | 497.598 | 494.840 | 472.774 |
| LR chi-square | 63.428 | 66.186 | 88.253 |
| LR chi-square p-value | <0.001 | <0.001 | <0.001 |
| McFadden R^2^ | 0.113 | 0.118 | 0.157 |
| McFadden adjusted R^2^ | 0.077 | 0.086 | 0.115 |
| Cox-Snell/ML R^2^ | 0.140 | 0.146 | 0.190 |
| Cragg Uhler/Nagelkerke R^2^ | 0.190 | 0.198 | 0.257 |
| Efron R^2^ | 0.141 | 0.148 | 0.196 |
| Tjur’s D | 0.142 | 0.149 | 0.196 |
| Count | 0.681 | 0.674 | 0.698 |
| Count (adjusted) | 0.178 | 0.160 | 0.221 |
| AIC | 517.598 | 512.840 | 496.774 |
| AIC divided by N | 1.232 | 1.221 | 1.183 |
| BIC | 558.000 | 549.202 | 545.257 |
