# Supplementary material for: Predictors of COVID-19 vaccination intention among students in Ghana: An application of the Health Belief Model and Theory of Planned Behaviour
Source: PLOS Glob Public Health. 2025 Dec 29;5(12):e0005561. doi: 10.1371/journal.pgph.0005561 (PMC12747339; doi:10.1371/journal.pgph.0005561)
Supplement: S3 Table — (DOCX) [file pgph.0005561.s004.docx]

| **SN** | **Variable Name** | **Category and Value Labels** |
| --- | --- | --- |
| 1 | Agegroup | RECODE of Age: 1 = 15-17, 2 = 18-30 |
| 2 | Sex1 | Sex: 1 = Female, 2 = Male |
| 3 | Religion1 | Religion: 1 = African Traditional Religion, 2 = Christianity, 3 = Islam |
| 4 | Ethnicgroup1 | Ethnic group: 1 = Akan, 2 = Buem, 3 = Ewe, 4 = Guan, 5 = Hausa, 6 = Konkomba, 7 = Others |
| 5 | Year1 | Year: 1 = SHS 2, 2 = SHS 3 |
| 6 | Course1 | Course: 1 = Business, 2 = Fashion Design, 3 = General Agriculture, 4 = General Arts, 5 = General Science, 6 = 6. Home Economics, 7 = 7. Technical, 8 = 8. Visual Art |
| 7 | course_cat | RECODE of Course: 0 = 0. Academic, 1 = Techn_Applied, 2 = Creat_Voca |
| 8 | studtype1 | Student type: 1 = Boarding student, 2 = Day student |
| 9 | HRC1 | Corona: 1 = No, 2 = Yes |
| 10 | HRC2 | Condition: 1 = No, 2 = Yes |
| 11 | HRC3 | Health status: 1 = No, 2 = Yes |
| 12 | AD1 | Face mask usage: 1 = No, 2 = Yes |
| 13 | AD3 | Handwashing: 1 = No, 2 = Yes |
| 14 | AD5 | Social distancing: 1 = No, 2 = Yes |
| 15 | AD7 | Hand sanitizer: 1 = No, 2 = Yes |
| 16 | AT1 | Tedious: 1 = Agree, 2 = Disagree, 3 = Neutral, 4 = Strongly Agree, 5 = Strongly Disagree |
| 17 | AT2 | Benefit: 1 = Agree, 2 = Disagree, 3 = Neutral, 4 = Strongly Agree |
| 18 | AT3 | Behavioral belief: 1 = Agree, 2 = Disagree, 3 = Neutral, 4 = Strongly Agree, 5 = Strongly Disagree |
| 19 | AT4 | Recommendation: 1 = Agree, 2 = Disagree, 3 = Neutral, 4 = Strongly Agree, 5 = Strongly Disagree |
| 20 | AT5 | Mark: 1 = Agree, 2 = Disagree, 3 = Neutral, 4 = Strongly Agree, 5 = Strongly Disagree |
| 21 | AT6 | Die in 2 years: 1 = Agree, 2 = Disagree, 3 = Neutral, 4 = Strongly Agree, 5 = Strongly Disagree |
| 22 | SN1 | Subjective norms (Friends): 1 = Agree, 2 = Disagree, 3 = Neutral, 4 = Strongly Agree, 5 = Strongly Disagree |
| 23 | SN2 | Approves: 1 = Agree, 2 = Disagree, 3 = Neutral, 4 = Strongly Agree, 5 = Strongly Disagree |
| 24 | SN3 | Relatives: 1 = Agree, 2 = Disagree, 3 = Neutral, 4 = Strongly Agree, 5 = Strongly Disagree |
| 25 | SN4 | Available: 1 = Agree, 2 = Disagree, 3 = Neutral, 4 = Strongly Agree, 5 = Strongly Disagree |
| 26 | SN5 | Teach: 1 = Agree, 2 = Disagree, 3 = Neutral, 4 = Strongly Agree, 5 = Strongly Disagree |
| 27 | PBC1 | Easily: 1 = Agree, 2 = Disagree, 3 = Neutral, 4 = Strongly Agree, 5 = Strongly Disagree |
| 28 | PBC2 | Most effective: 1 = Agree, 2 = Disagree, 3 = Neutral, 4 = Strongly Agree, 5 = Strongly Disagree |
| 29 | PS1 | Suffer: 1 = Agree, 2 = Disagree, 3 = Neutral, 4 = Strongly Agree, 5 = Strongly Disagree |
| 30 | PS2 | Likely to recover: 1 = Agree, 2 = Disagree, 3 = Neutral, 4 = Strongly Agree, 5 = Strongly Disagree |
| 31 | PS3 | Dying: 1 = Agree, 2 = Disagree, 3 = Neutral, 4 = Strongly Agree, 5 = Strongly Disagree |
| 32 | PS4 | Hospitalized: 1 = Agree, 2 = Disagree, 3 = Neutral, 4 = Strongly Agree, 5 = Strongly Disagree |
| 33 | PSU1 | Current: 1 = Agree, 2 = Disagree, 3 = Neutral, 4 = Strongly Agree, 5 = Strongly Disagree |
| 34 | PSU2 | Future: 1 = Agree, 2 = Disagree, 3 = Neutral, 4 = Strongly Agree, 5 = Strongly Disagree |
| 35 | PSU3 | Family: 1 = Agree, 2 = Disagree, 3 = Neutral, 4 = Strongly Agree, 5 = Strongly Disagree |
| 36 | PSU4 | Friends: 1 = Agree, 2 = Disagree, 3 = Neutral, 4 = Strongly Agree, 5 = Strongly Disagree |
| 37 | PB1 | Decrease: 1 = Agree, 2 = Disagree, 3 = Neutral, 4 = Strongly Agree, 5 = Strongly Disagree |
| 38 | PB2 | Academic: 1 = Agree, 2 = Disagree, 3 = Neutral, 4 = Strongly Agree, 5 = Strongly Disagree |
| 39 | PB3 | Suffering: 1 = Agree, 2 = Disagree, 3 = Neutral, 4 = Strongly Agree, 5 = Strongly Disagree |
| 40 | PBR1 | Expensive: 1 = Agree, 2 = Disagree, 3 = Neutral, 4 = Strongly Disagree |
| 41 | PBR2 | Effort: 1 = Agree, 2 = Disagree, 3 = Neutral, 4 = Strongly Disagree |
| 42 | PBR3 | Effect: 1 = Agree, 2 = Disagree, 3 = Neutral, 4 = Strongly Disagree |
| 43 | PBR4 | NID: 1 = Agree, 2 = Disagree, 3 = Neutral, 4 = Strongly Disagree |
| 44 | PBR5 | Infertility: 1 = Agree, 2 = Disagree, 3 = Neutral, 4 = Strongly Disagree |
| 45 | PBR6 | Kill: 1 = Agree, 2 = Disagree, 3 = Neutral, 4 = Strongly Disagree |
| 46 | SE1 | Precaution: 1 = Agree, 2 = Disagree, 3 = Neutral, 4 = Strongly Agree, 5 = Strongly Disagree |
| 47 | SE2 | On time: 1 = Agree, 2 = Disagree, 3 = Neutral, 4 = Strongly Agree, 5 = Strongly Disagree |
| 48 | SE3 | Difficult: 1 = Agree, 2 = Disagree, 3 = Neutral, 4 = Strongly Agree, 5 = Strongly Disagree |
| 49 | CA1 | Media: 1 = Agree, 2 = Disagree, 3 = Neutral, 4 = Strongly Agree, 5 = Strongly Disagree |
| 50 | CA2 | Family: 1 = Agree, 2 = Disagree, 3 = Neutral, 4 = Strongly Agree, 5 = Strongly Disagree |
| 51 | CA3 | Teachers: 1 = Agree, 2 = Disagree, 3 = Neutral, 4 = Strongly Agree, 5 = Strongly Disagree |
| 52 | CA4 | Problem: 1 = Agree, 2 = Disagree, 3 = Neutral, 4 = Strongly Agree, 5 = Strongly Disagree |
| 53 | CA5 | Info: 1 = Agree, 2 = Disagree, 3 = Neutral, 4 = Strongly Agree, 5 = Strongly Disagree |
| 54 | Perce_Suscep | Perceived Susceptibility: Numeric (float), Range [4,20] |
| 55 | Perce_Seve | Perceived Severity: Numeric (float), Range [5,20] |
| 56 | Perce_Bene | Perceived Benefits: Numeric (float), Range [3,15] |
| 57 | Perce_Bar | Perceived Barriers: Numeric (float), Range [6,24] |
| 58 | Cues_Act | Cues to Action: Numeric (float), Range [5,25] |
| 59 | Attitude | Attitude: Numeric (float), Range [7,28] |
| 60 | Sub_Norms | Subjective Norms: Numeric (float), Range [5,25] |
| 61 | Perce_Behavior | Perceived Behavior: Numeric (float), Range [2,10] |
| 62 | Self_Effi | Self-Efficacy: Numeric (float), Range [3,15] |
| 63 | intention | RECODE of intent (willing): 0 = No, 1 = Yes |
