# Supplementary material for: Predictors of COVID-19 vaccination intention among students in Ghana: An application of the Health Belief Model and Theory of Planned Behaviour
Source: PLOS Glob Public Health. 2025 Dec 29;5(12):e0005561. doi: 10.1371/journal.pgph.0005561 (PMC12747339; doi:10.1371/journal.pgph.0005561)
Supplement: Attachment — Submitted filename: Reviewed_PGPH-D-25-03045_reviewer.pdf [file pgph.0005561.s005.pdf]

PLOS Global Public Health

Predictors of COVID-19 vaccination intention using Health Belief Model and Theory of Planned Behaviour: A cross-sectional study in Jasikan Municipality, Ghana

--Manuscript Draft--

**Manuscript Number:**	PGPH-D-25-03045
**Article Type:**	Research Article
**Full Title:**	Predictors of COVID-19 vaccination intention using Health Belief Model and Theory of Planned Behaviour: A cross-sectional study in Jasikan Municipality, Ghana
**Short Title:**	Predictors of COVID-19 vaccination intention in Jasikan Municipality, Ghana
**Corresponding Author:**	Mawuli Gohoho, M.Phil<br>GHS: Ghana Health Service<br>Jasikan, GHANA
**Order of Authors:**	Hudatu Ahmed, BPH
	Mawuli Gohoho, M.Phil
	Samuel Adolf Bosoka, M.Phil
	George Sarpong Agyemang, M.Phil
	Sorengmen Amos Ziema, M.Phil
	James Alorwu, M.Phil
	Isaac Annobil, MPH
	Veronica Okwuchi Charles-Unadike, PhD
**Keywords:**	COVID-19 vaccines; vaccination; intention; health belief model; theory of planned behaviour; students; Ghana
**Abstract:**	Background: In 2021, most COVID-19 cases in Jasikan Municipality of Ghana were among students, putting them at risk of severe clinical outcomes that could negatively affect their academic performance. Schools can serve as environments that promote transmission of infectious diseases due to close contact among students. However, students can also influence community health behaviours through peer and family interactions which makes their vaccination decisions important for broader community protection. Limited evidence exists on factors influencing their decisions regarding the uptake of COVID-19 vaccines. This study therefore examined predictors of students' intention to vaccinate against COVID-19 using the Health Belief Model (HBM) and Theory of Planned Behaviour (TPB) to inform strategies to improve vaccine acceptance within Jasikan Municipality, Ghana.<br>Methods: The study used a cross-sectional design conducted from 25 July to 5 August 2022 and a multistage sampling technique was used to select students from Senior High Schools. We used a pilot-tested questionnaire to collect data on socio-demographic, COVID-19 protocol adherence, health-related, HBM and TPB constructs, and vaccination intention. Descriptive and inferential statistics were performed. Hierarchical logistic regression was used to assess the predictors of COVID-19 vaccination intention with statistical significance set at P <0.05.<br>Results: Of 420 students, 206 (49.0%) were females and 141 (33.6%) aged 15-17 years. Two hundred and fifty-seven (61.0%) students expressed the intention to vaccinate against COVID-19. Previous experience of COVID-19 (AOR = 3.26; 95% CI: 1.11 - 9.56), adherence to social distancing (AOR = 1.85; 95% CI: 1.17 - 2.91), attitude towards vaccination (AOR = 1.10; 95% CI: 1.03 - 1.18), subjective norms (AOR = 1.09; 95% CI: 1.04 - 1.15), and self-efficacy (AOR = 1.20; 95% CI: 1.11 - 1.31) were significant predictors of COVID-19 vaccination intention.<br>Conclusion: Six out of ten students intended to vaccinate against COVID-19 in Jasikan. This intention was predicted by prior COVID-19 experience, social distancing behaviour, and key constructs from the HBM and TPB. We recommend intensifying interventions that target attitudes, social influences, and students' confidence in their ability to get vaccinated to strengthen vaccine acceptance.

Powered by Editorial Manager® and ProduXion Manager® from Aries Systems Corporation

Additional Information:	
Question	Response
**Financial Disclosure**<br><br>Enter a financial disclosure statement that describes the sources of funding for the work included in this submission and the role the funder(s) played. This includes grants and any commercial funding of the work or authors.<br><br>This statement will be typeset if the manuscript is accepted for publication.<br><br>*Please review the submission guidelines and the instructions link below for detailed requirements and guidance.*	The author(s) received no specific funding for this work.
**Competing Interests**<br><br>On behalf of all authors, disclose any competing interests that could be perceived to bias this work.<br><br>This statement will be typeset if the manuscript is accepted for publication.<br><br>*Please review the instructions link below and PLOS Global Public Health's competing interests policy to determine what information must be disclosed at submission.*	The authors have declared that no competing interests exist.
**Data Availability**<br><br>Before publication, Authors are required to make fully available and without restriction all data underlying their findings. Please see our PLOS Data Policy page for detailed information on this policy.<br><br>A **Data Availability Statement**, detailing	All data supporting the findings of this study are fully contained within the manuscript.

Powered by Editorial Manager® and ProduXion Manager® from Aries Systems Corporation

where the data can be accessed, is required at first submission. Insert your Data Availability Statement in the box below.<br><br>Please see the data reporting section of our submission guidelines for instructions on what you need to include in your Data Availability Statement.<br><br>This statement will be typeset if the manuscript is accepted for publication.<br><br>*PLOS allows rare exemptions to address legal and ethical concerns. If you have legal or ethical restrictions, please use the box below to detail these in full sentences for the Journal team to consider.*	

Powered by Editorial Manager® and ProduXion Manager® from Aries Systems Corporation

Manuscript Click here to access/download;Manuscript;Intention_Manuscript

Predictors of COVID-19 vaccination intention using Health Belief Model and Theory of Planned Behaviour: A cross-sectional study in Jasikan Municipality, Ghana

Hudatu Ahmed[1], Mawuli Gohoho[2,7*], Samuel Adolf Bosoka[3,7], George Sarpong Agyemang[4,7], Sorengmen Amos Ziema[5,7], James Alorwu[6,7], Isaac Annobil[2], Veronica Okwuchi Charles-Unadike[1]

[1]Fred N. Binka School of Public Health, University of Health and Allied Sciences, Hohoe, Volta Region, Ghana

[2]Jasikan Municipal Health Directorate, Ghana Health Service, Jasikan, Oti Region, Ghana

[3]Disease Surveillance Unit, Volta Regional Health Directorate, Ghana Health Service, Ho, Volta Region, Ghana

[4]Department of Public Health, Catholic University of Ghana, Fipre, Ghana

[5]Department of Health Information and Records Management, Ho Teaching Hospital, Ho, Volta Region, Ghana

[6]Tumu Municipal Hospital, Ghana Health Service, Tumu, Upper West Region, Ghana

[7]Ghana Field Epidemiology and Laboratory Training Programme, Accra, Ghana

*Corresponding author

Email: mawuli.gohoho@ghs.gov.gh (MG)

Background: In 2021, most COVID-19 cases in Jasikan Municipality of Ghana were among students, putting them at risk of severe clinical outcomes that could negatively affect their academic performance. Schools can serve as environments that promote transmission of infectious diseases due to close contact among students. However, students can also influence community health behaviours through peer and family interactions which makes their vaccination decisions important for broader community

protection. Limited evidence exists on factors influencing their decisions regarding the uptake of COVID-19 vaccines. This study therefore examined predictors of students' intention to vaccinate against COVID-19 using the Health Belief Model (HBM) and Theory of Planned Behaviour (TPB) to inform strategies to improve vaccine acceptance within Jasikan Municipality, Ghana.

Methods: The study used a cross-sectional design conducted from 25 July to 5 August 2022 and a multistage sampling technique was used to select students from Senior High Schools. We used a pilot-tested questionnaire to collect data on socio-demographic, COVID-19 protocol adherence, health-related, HBM and TPB constructs, and vaccination intention. Descriptive and inferential statistics were performed. Hierarchical logistic regression was used to assess the predictors of COVID-19 vaccination intention with statistical significance set at $P < 0.05$.

Results: Of 420 students, 206 (49.0%) were females and 141 (33.6%) aged 15-17 years. Two hundred and fifty-seven (61.0%) students expressed the intention to vaccinate against COVID-19. Previous experience of COVID-19 (AOR = 3.26; 95% CI: 1.11 - 9.56), adherence to social distancing (AOR = 1.85; 95% CI: 1.17 - 2.91), attitude towards vaccination (AOR = 1.10; 95% CI: 1.03 - 1.18), subjective norms (AOR = 1.09; 95% CI: 1.04 - 1.15), and self-efficacy (AOR = 1.20; 95% CI: 1.11 - 1.31) were significant predictors of COVID-19 vaccination intention.

Conclusion: Six out of ten students intended to vaccinate against COVID-19 in Jasikan. This intention was predicted by prior COVID-19 experience, social distancing behaviour, and key constructs from the HBM and TPB. We recommend intensifying interventions that target attitudes, social influences, and students' confidence in their ability to get vaccinated to strengthen vaccine acceptance.

Keywords: COVID-19 vaccines; vaccination; intention; health belief model; theory of planned behaviour; students; Ghana

Introduction

In public health, vaccinations represent a major advancement, preventing millions of deaths annually including those related to the COVID-19 pandemic [1,2]. In response to the rapid global spread of COVID-19, the World Health Organisation (WHO) declared it a public health emergency of international concern on 30 January 2020 [3]. The pandemic subsequently affected over 770 million people globally, resulting in more than 7 million deaths and significant disruptions to healthcare systems and economies [4].

In the Ghanaian context, the first two confirmed COVID-19 cases were reported on 12 March 2020, after two travellers returned from Norway and Turkey [5,6]. Following this, the government of Ghana instituted certain travel limitations, social distancing measures along with guidelines for good handwashing practices and wearing of nose marks to help contain the situation [6]. All educational institutions, including both public and private senior high schools (SHS), were closed [5]. As of 3 January 2021, the Government of Ghana announced the re-opening of academic institutions to allow all school children and students to return [5]. On 24 February 2021, Ghana received COVAX vaccine doses and rolled out a national vaccination programme that included the President of the Republic receiving the first injection televised on national television to encourage and promote uptake among the general populace [7,8] . In fact, it is worth noting that, as of 31 December 2023, over 25 million doses of the COVAX vaccine had been administered, however, only 35% of the population vaccinated with a complete primary series [9].

Despite the essential role of vaccines, vaccine hesitancy which is defined as the delay in acceptance or refusal of vaccination despite the availability of vaccination services has emerged as a significant global health threat, recognized by the WHO even before

the COVID-19 pandemic [10,11]. This reluctance is often attributed to various factors, including concerns about vaccine safety and efficacy, which were amplified by the unprecedented speed of COVID-19 vaccine development [12–14]. Misinformation and unfounded rumours on social media also contribute significantly to public mistrust [14–16]. For instance, past incidents in Nigeria and Kenya involving polio vaccine boycotts due to false claims demonstrates the profound impact of misinformation on vaccination campaign [17–19]. Studies have documented fear of contracting an infection from the vaccine, concerns about side effects, beliefs about microchips or infertility, underestimation of disease impact, and a general lack of trust in government officials, state institutions, and pharmaceutical companies as common reasons for COVID-19 vaccine hesitancy [14,20–22]. Based on this, some would actually prefer resorting to traditional remedies or hold firm onto their spiritual or religious belief that discourages vaccination [14,21].

Students constitute a vital and distinct demographic group within the population [23–26]. They act as potential "messengers" in disseminating effective health-promoting behaviours to their families and communities [23,27]. The COVID-19 pandemic significantly affected students physically, academically, financially, and psychologically as well [28,29]. The environment within which students find themselves and their social interactions might fuel outbreaks of COVID-19 disease in those localised settings [23,30]. For instance, in the Jasikan Municipality, information from the 2021 Jasikan District Annual Health Report revealed that 54.5% of the COVID-19 cases were among students. This suggests a high incidence of COVID-19 in the student populace, which might increase the chances of complications, severe clinical outcomes, and possibly affect their academic outputs as observed in previous studies [29,31].

In response to such trends and as part of measures to increase protection among adolescents, the Pfizer-BioNTech COVID-19 vaccine was recommended for use in persons aged 15 years and above in schools to help halt the transmission of COVID-19 and increase the level of protection against the virus. The Ghana Health Service and the Food and Drugs Authority granted approval for extending the use of the vaccine to cover children from 15 years and above. This recommendation was communicated in a letter dated 19 November 2021, from the Director-General of the Ghana Technical and Vocational Education Training (TVET) Service, addressed to all Heads of TVET institutions (Public and Private), requesting them to facilitate the vaccination of all learners aged 15 or older in their institutions across the country.

However, the current study was conceptualised prior to the release of this national directive, in recognition of the significant burden of COVID-19 among students and the need to obtain relevant information regarding their intention to take up the vaccine in order to develop targeted health promotion interventions. In Jasikan, limited evidence exists on factors influencing students' decisions regarding the uptake of COVID-19 vaccines. Based on this rationale, the study adopted behavioural theories, including the Health Belief Model (HBM) and the Theory of Planned Behaviour (TPB) to examine their intentions to vaccinate against COVID-19. HBM focuses on individual beliefs about health conditions and behaviours, explaining health-promoting actions based on perceived susceptibility, severity, benefits, barriers, cues to action, and self-efficacy. It predicts preventive health behaviours by assessing how people perceive threats and the value of taking recommended action [32–34]. TPB, on the other hand, emphasises behavioural intention as the most direct predictor of action [35]. It integrates attitude toward the behaviour, subjective norms, and perceived behavioural control, capturing both individual motivation and perceived ability to act [35,36]. Integrating both models

account for both cognitive evaluations and social-influence pathways shaping vaccination decisions.

This study therefore assessed the predictors of students' intention to vaccinate against COVID-19 using the comprehensive frameworks of HBM and TPB in the Jasikan Municipality of Ghana. Based on these factors identified among students, improved vaccination strategies can be proposed to increase uptake and protect this COVID-19 susceptible group. Identifying strategies that are more acceptable to students may improve vaccine acceptance and expand coverage in future vaccine rollout.

Methods

Ethics statement

Ethical approval was obtained from the University of Health and Allied Sciences Research Ethics Committee (UHAS-REC A.10 [23] 21-22). Permission was also granted by the Jasikan District Office, Ghana Education Service, to access the schools for data collection. Participation was voluntary. Written informed consent was obtained from participants aged 18 years and above, while assent was obtained from those below 18, with additional consent from school authorities. The study purpose, risks, and benefits were clearly explained to all participants. There were no known risks or direct benefits. No compensation was provided, though participants were appreciated for their time. Data were anonymised, stored securely with password protection on a personal laptop accessible only to the principal investigator. Participants could withdraw at any point without consequence.

Study site

The study was conducted in four senior high schools (SHS): Bueman SHS, Okadjakrom Senior High Technical School (SHTS), Father Dogli SHS, and Baglo Ridge SHS, all located in the Jasikan Municipality in the Oti Region of Ghana. The municipality shares boundaries with the Kadjebi District to the north, the Biakoye District to the west, the Guan District to the south, and the Republic of Togo to the east. According to the 2021 Jasikan District Annual Health Report, approximately 54.5% of the total COVID-19 cases were among students in the municipality.

Study design and population

A cross-sectional study was conducted among SHS students in Jasikan Municipality, grounded in the HBM and TPB as applied in an Israeli study [37]. The study population included students aged 15 years and above who were present during the data collection period. The inclusion of students aged 15 years and above was based on the national guideline recommending COVID-19 vaccine for use among persons in this age group in schools in Ghana. However, those who were severely ill, absent, or had mental disabilities were excluded to ensure participants could provide informed responses and actively participate in the study without compromising their wellbeing or data quality.

Sample size and sampling method

The sample size was computed using the single population proportion formula [38], $n = \frac{(z_{\alpha/2})^2 \times p(1-p)}{d^2}$ with the following assumptions: an estimated proportion of persons aged 15 years and above with the intention to vaccinate against COVID-19 in Ghana (p = 51%) [39]; Confidence level at 95% (Zα/2) = 1.96, a margin of error (d) = 0.05 and non-response rate = 10%. The minimum sample size determined was 420. To ensure a representative and comparability, a multistage sampling procedure was employed in the selection of study participants. At stage one, all the four SHSs were involved. At stage two, the students from the schools were stratified into SHS 2 and SHS 3 to have 8 strata from the four schools. During the period of data collection, only those in SHS 2 and SHS 3 were available. In the third stage, the number of students to be selected from each stratum was determined proportionate to the stratum population size. In the fourth stage, simple random sampling was applied within each stratum to select eligible participants to have 420 students.

Data collection procedure

To obtain the responses from the participants, a pretested semi-structured questionnaire was used to collect the data from 25 July to 5 August 2022. Amidst the COVID-19 pandemic, trained research assistants were educated on prevention and provided with face masks and hand sanitisers to reduce the probability of person-to-person transmission. Having explained the purpose of the study to the participants and obtaining consent, research assistants distributed the questionnaires to the participants in the school visited. This questionnaire had sections on sociodemographic characteristics, health-related variables, COVID-19 protocol adherence, HBM, TPB and COVID-19 vaccination intention. The reliability of the HBM and TPB constructs assessed using Cronbach's alpha ranged from 0.63 to 0.86 which was based on the dataset obtained for the study (Table 1).

Variables and measurements

The dependent variable was the intention to vaccinate against COVID-19, initially assessed using a single-item question on a 5-point Likert scale (1 = Certain, 2 = Very Likely, 3 = Somewhat Likely, 4 = Not Likely, 5 = Never). This variable was dichotomised into a binary outcome (1 = Intends to get vaccinated, 0 = Does not intend to get vaccinated) to facilitate comparative analyses between those with the intention to vaccinate and those who do not, with values 1–3 coded as 1 and 4–5 as 0.

The independent variables were organized into five blocks:

- **Socio-demographic:** included (1) age (categorised into 15–17 and 18–30 years from an original numeric variable to examine age group differences), (2) gender, (3) religious affiliation, (4) ethnic group, (5) school attending, (6) academic level, (7) residency status and (8) course of study (recategorised into a new variable with three groups: 1 = academic [reference group: business,

general arts, general science], 2 = technical/applied [technical, general agriculture, home economics], 3 = creative/vocational [fashion design, visual art]) (Fig 1).

- **Health-related:** comprised (1) history of COVID-19, (2) family history of underlying disease conditions, and (3) perception of good health (Fig 1).
- **COVID-19 protocol adherence:** included (1) face mask usage, (2) adherence to handwashing, (3) adherence to social distancing practices and (4) hand sanitiser usage (Fig 1).
- **HBM constructs** (see Table 1): consisted of (1) perceived susceptibility (four items, Cronbach's α = 0.82), (2) perceived severity (four items, Cronbach's α = 0.66), (3) perceived benefits (three items, Cronbach's α = 0.74), (4) perceived barriers (six items, Cronbach's α = 0.75), (5) self-efficacy (three items, Cronbach's α = 0.63) and (6) cues to action (five items, Cronbach's α = 0.79).
- **TPB constructs** (see Table 1): included (1) attitude (six items, Cronbach's α = 0.69), (2) subjective norms (five items, Cronbach's α = 0.86), (3) and perceived behavioural control (PBC) (two items, Cronbach's α = 0.69)

Items in the HBM and TPB were measured on a 5-point Likert scale ranging from 1 (strongly disagree) to 5 (strongly agree). Negatively worded items were reverse-scored. Mean scores were computed for each item to derive the independent HBM and TPB constructs (Table 1).

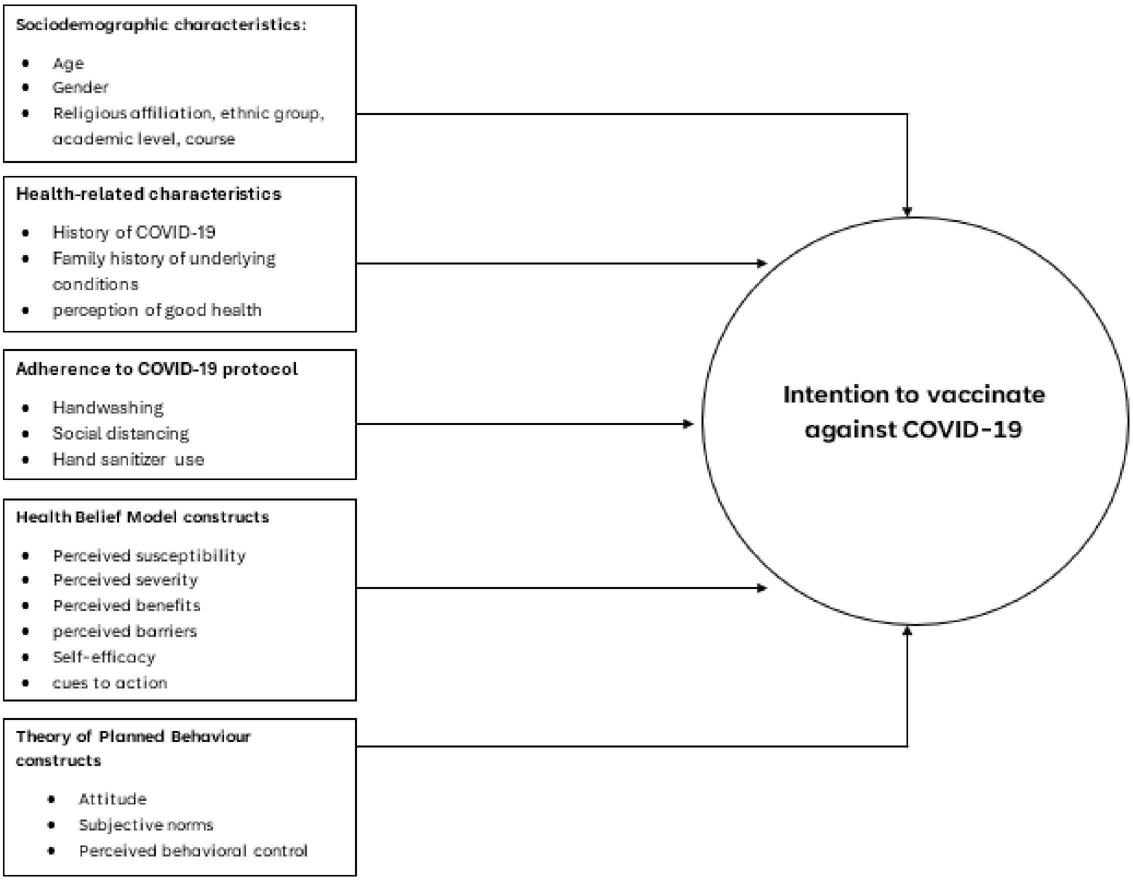

Fig 1: Conceptual framework of predictors of intention to vaccinate against COVID-19

Table 1: Reliability for HBM and TPB constructs related to COVID-19 vaccination intention

Measures	Items	Mean	std	Cronbach's Alpha (α)
**Model 1: Health Belief Model**				
Perceived Severity	If I should get infected with COVID-19…...<br>• I do not think it will cause too much suffering<br>• the chance of recovering from it is very high<br>• the chance of dying is very high<br>• I can be hospitalised for weeks	13.73	2.52	0.66
Perceived Susceptibility	I believe that if I do not get vaccinated the chance of…<br>• getting COVID-19 currently is high<br>• getting COVID-19 in future is high<br>• my family getting COVID-19 is high<br>• my friends getting COVID-19 is high	11.72	4.36	0.82
Perceived Benefit	I believe that getting vaccinated against COVID-19…<br>• will decrease my chances of getting COVID-19<br>• will improve my health and improve my academics<br>• will prevent suffering and complications of COVID-19	10.41	3.18	0.74
Perceived Barrier	• Going to get vaccinated is expensive<br>• Getting vaccinated requires time and effort<br>• The COVID-19 vaccine can cause some reaction to my body<br>• I do not have a national ID for the vaccination<br>• The COVID-19 vaccine can cause infertility<br>• The COVID-19 vaccine can kill	13.24	4.41	0.75
Self-Efficacy	• If I take all the necessary precautions (handwashing, face mask usage etc.), I do not need to be vaccinated against COVID-19<br>• I can receive the COVID-19 vaccine on time<br>• It is not difficult for me to receive the COVIID-19 vaccine	9.92	2.94	0.63
Cues to Action	• The chances of me getting vaccinated against COVID-19 will increase if…<br>• celebrities and famous people on social media express support for the benefit of the vaccine<br>• friends and family express support for the benefit of the vaccine<br>• my teachers and headteachers advise me to take it<br>• it does not cause serious problems to vaccinated people<br>• I get complete and credible<br>• information on the vaccine	16.46	4.65	0.79
**Model 2: Theory of planned behavior**				
Attitude	• Getting vaccinated is a tedious process that requires time and effort<br>• COVID-19 vaccine is beneficial for the students	19.41	3.69	0.69

**Measures**	**Items**	**Mean**	**std**	**Cronbach's Alpha (α)**
	• COVID-19 vaccine is effective in preventing the disease<br>• Once a COVID-19 vaccine is recommended, getting it would be good<br>• COVID-19 vaccine is the mark of the beast (666)<br>• People will die from taking the COVID-19 vaccine after 2 years			
Subjective Norms	• Most of my friends will take the COVID-19 vaccine<br>• My family who are important to me would approve of me getting any available COVID-19 vaccine<br>• My relatives who are important to me would approve of me getting any available COVID-19 vaccine<br>• My friends who are important to me would approve of me getting any available COVID-19 vaccine<br>• My headmaster/teachers who are important to me would approve of me getting any available COVID-19 vaccine	16.51	4.97	0.86
Perceived Behavioural Control	• I could easily receive a COVID-19 vaccine if I wanted to<br>• It is mostly up to me whether or not I have COVID-19 vaccination	7.36	2.09	0.69

Data management and analysis

Data were checked for completeness and consistency before entry into EpiData version 3.1 and exported to STATA version 16 for analysis. Descriptive analysis involved computation of means, standard deviations, frequencies, and proportions to summarise key variables. Chi-square tests assessed associations between categorical variables and intention to vaccinate against COVID-19, while independent sample t-tests compared mean scores of HBM and TPB constructs between students based on vaccination intention. Effect sizes were estimated using Cohen's d and interpreted according to Cohen's benchmarks [40], where 0.20 indicates a small effect, 0.50 a moderate effect, and 0.80 a large effect. Negative values of d showed the direction of group differences, while interpretation was based on absolute values.

Hierarchical logistic regression analysis was conducted in three steps to identify predictors of vaccination intention. Model 1 included sociodemographic, health-related, adherence, and HBM variables. Model 2 included the same predictors, replacing HBM variables with TPB constructs. Model 3 combined all variables from the previous models. Sociodemographic variables were adjusted for in all models. HBM and TPB constructs were treated as continuous variables. Adjusted odds ratios (AOR) with 95% confidence intervals were reported. Variables included in the multivariable models were selected based on statistical significance from univariate analysis ($p < 0.05$). Model fit was evaluated using Cragg and Uhler's R^2 to estimate the proportion of variance in vaccination intention explained by the predictors.

Results

Students' characteristics: A total of 420 SHS students participated in the study with a response rate of 100%. Of these, 206 (49.0%) were female and 214 (51.0%) were male. Most of the students were aged 18–30 years [279 (66.4%)], while 141 (33.6%) were aged 15–17 years. Majority identified as Christians [364 (86.7%)], followed by Muslims [52 (12.4%)] and African Traditional Religion adherents [4 (1.0%)]. The predominant ethnic group was Ewe [163 (38.8%)], followed by Buem [74 (17.6%)], Guan [60 (14.3%)], Konkomba [49 (11.7%)] and Akan [45 (10.7%)]. Regarding school attended, 90 (21.4%) were from Baglo Ridge SHS, 108 (25.7%) from Bueman SHS, 117 (27.9%) from Father Dogli Memorial SHS, and 105 (25.0%) from Okadjakrom SHTS. Most participants were in SHS 2 [248 (59.0%)], followed by SHS 3 [172 (41.0%)]. The academic programmes pursued by participants included General Arts [148 (35.2%)], Technical [135 (32.1%)], Home Economics [60 (14.3%)], General Science [54 (12.9%)], and Business [23 (5.5%)]. A total of 364 (86.7%) participants resided in school, while 56 (13.3%) were non-resident students (Table 2).

Health-related characteristics: Only 26 (6.2%) of the participants reported ever having COVID-19, while 394 (93.8%) had not. A family history of underlying conditions was reported by 41 (9.8%) participants. Most students [308 (73.3%)] perceived their health status as good (Table 2).

Adherence to COVID-19 protocols: Mask usage was reported by 275 (65.5%) of the participants. Three hundred and sixty-nine of the participants [369 (87.9%)] practiced regular handwashing, 333 (79.3%) reported hand sanitiser use, and 275 (65.5%) practiced social distancing (Table 3).

Intention to vaccinate against COVID-19: Out of the total participants, 257 [61.2% (56.4% - 65.8%)] expressed an intention to receive the COVID-19 vaccine, while 163 [38.8% (34.2% - 43.6%)] did not (Fig 2).

Univariate analysis: For the univariate analysis between sociodemographic variables and intention to vaccinate against COVID-19, age group was significantly associated with intention to vaccinate (χ^2 = 3.97, p = 0.046). Academic programme was also significantly associated with vaccination intention (χ^2 = 18.01, p = 0.012). Among the health-related variables, history of COVID-19 showed a statistically significant association (χ^2 = 7.25, p = 0.007). Handwashing practice (χ^2 = 5.40, p = 0.02) and social distancing (χ^2 = 10.15, p = 0.001) were significantly associated with intention to vaccinate (Table 2).

For the HBM variables, the mean perceived susceptibility was significantly higher among participants intending to vaccinate (t = -1.99, p = 0.047), with a small effect size (Cohen's d = -0.20). The mean perceived benefit was also significantly higher among those intending to vaccinate (t = -3.60, p = 0.0004), with a small effect size (Cohen's d = -0.36). Further, the mean self-efficacy was significantly higher among those intending to vaccinate (t = -5.05, p < 0.0001, Cohen's d = -0.51) with moderate effect size. No significant differences were observed for mean perceived severity, barriers, or cues to action. Among TPB constructs, the mean attitude score was significantly higher among those with intention to vaccinate (t = -5.47, p < 0.0001, Cohen's d = -0.55) with moderate effect size. Similarly, those with intention to vaccinate reported significantly higher mean subjective norms (t = -5.35, p < 0.0001, Cohen's d = -0.54) with moderate effect size. No significant difference was found in perceived behavioural control between the two groups (Table 3).

Factors associated with intention to vaccinate against COVID-19: In Model 1, which included sociodemographic, health-related, adherence, and HBM constructs, students' intention to vaccinate was significantly associated with history of COVID-19 infection (AOR = 3.55; 95% CI: 1.25 to 10.12; p = 0.018), adherence to social distancing (AOR = 1.94; 95% CI: 1.25 to 3.02; p = 0.003), perceived benefit (AOR = 1.11; 95% CI: 1.03 to 1.20; p = 0.006), and self-efficacy (AOR = 1.21; 95% CI: 1.12 to 1.30; $p < 0.001$) (Table 4). The model explained 19% of the variance in vaccination intention (Cragg & Uhler's $R^2 = 0.19$) (See S1 Table).

In Model 2, which replaced the HBM constructs with TPB constructs, history of COVID-19 infection (AOR = 2.91; 95% CI: 1.03 to 8.21; p = 0.043), adherence to social distancing (AOR = 1.73; 95% CI: 1.11 to 2.69; p = 0.015), and being in a technical or applied course (AOR = 1.71; 95% CI: 1.07 to 2.73; p = 0.025) were significant. The TPB constructs including attitude (AOR = 1.13; 95% CI: 1.06 to 1.20; $p < 0.001$) and subjective norms (AOR = 1.08; 95% CI: 1.03 to 1.13; p = 0.001) were also significant (Table 4). The model explained 19.8% of the variance in vaccination intention (Cragg & Uhler's $R^2 = 0.198$) (See S1 Table).

In Model 3, which combined HBM and TPB constructs with other predictors, the model explained 25.7% of the variance in COVID-19 vaccination intention (Cragg & Uhler's $R^2 = 0.257$) (See S1 Table). Significant associations remained for history of COVID-19 infection (AOR = 3.26; 95% CI: 1.11 to 9.56; p = 0.032), adherence to social distancing (AOR = 1.85; 95% CI: 1.17 to 2.91; p = 0.008), attitude (AOR = 1.10; 95% CI: 1.03 to 1.18; p = 0.004), subjective norms (AOR = 1.09; 95% CI: 1.04 to 1.15; p = 0.001), and self-efficacy (AOR = 1.20; 95% CI: 1.11 to 1.31; $p < 0.001$). Perceived susceptibility and perceived benefit were not significant in the final model. The

technical or applied course variable was no longer significant at the 5% level (Table 4).

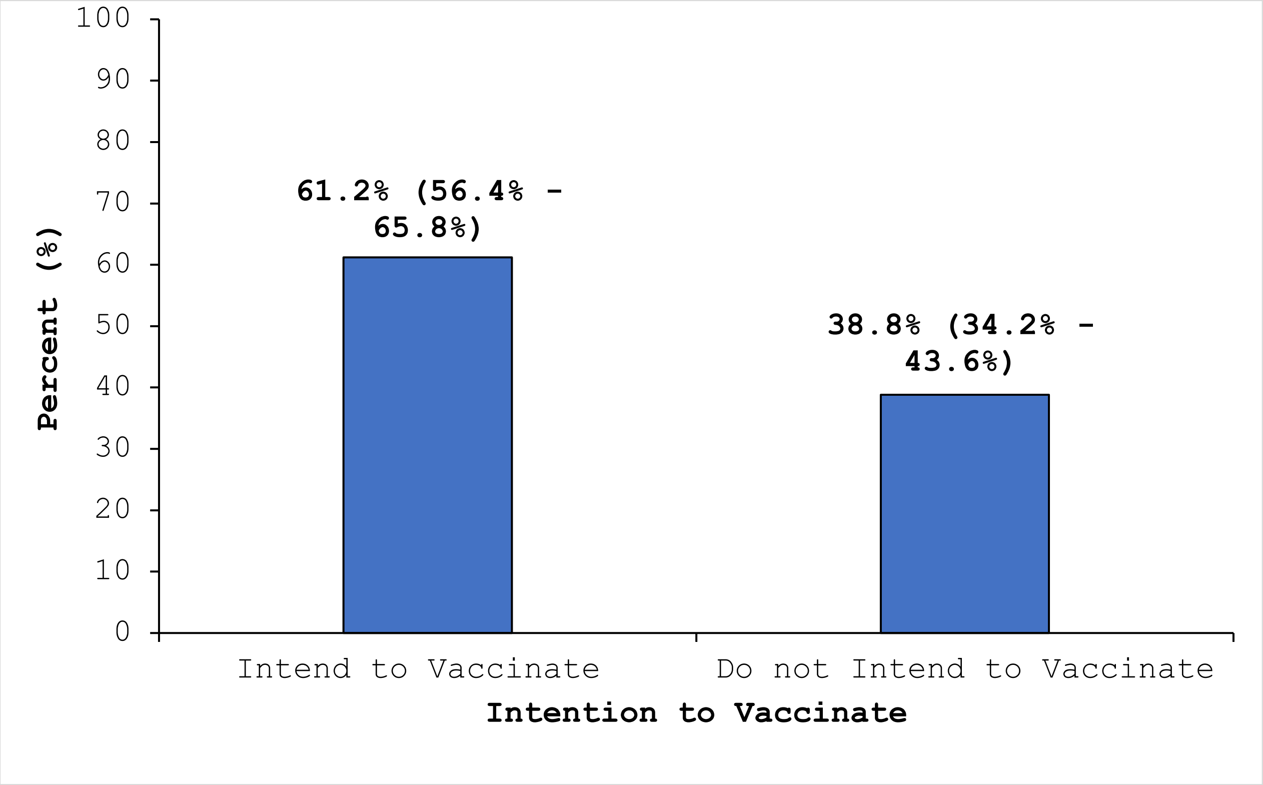

Fig 2: Proportion of students expressing Intention to vaccinate against COVID-19 in Jasikan Municipality, July to August 2022.

Table 2: Sociodemographic, health-related characteristics, and adherence to COVID-19 preventive protocols

Variables	Intention to vaccinate against COVID-19 No = 163 (38.8%) n (%)		Yes = 257 (61.2%) n (%)		Total = 420 (100%) n (%)		Chi-square (x^2)	p-value
**Demographics**								
**Age mean ± std dev.**	**17.89 ± 1.62**		**18.35 ± 1.89**		**18.21 ± 1.79**			
**Age group (years)**							2.38	0.123
15-17	62	(38.0)	79	(30.7)	141	(33.6)		
18-30	101	(62.0)	178	(69.3)	279	(66.4)		
**Sex**							3.97	**0.046***
Female	70	(42.9)	136	(52.9)	206	(49.0)		
Male	93	(57.1)	121	(47.1)	214	(51.0)		
**Religious affiliation**							2.74	0.255
Christianity	144	(88.3)	220	(85.6)	364	(86.7)		
Islam	19	(11.7)	33	(12.8)	52	(12.4)		
African Traditional Religion	0	(0.0)	4	(1.6)	4	(1.0)		
**Ethnic group**							6.22	0.399
Akan	15	(9.2)	36	(14.0)	51	(12.1)		
Buem	32	(19.6)	42	(16.3)	74	(17.6)		
Ewe	71	(43.6)	92	(35.8)	163	(38.8)		
Guan	6	(3.7)	13	(5.1)	19	(4.5)		
Hausa	16	(9.8)	24	(9.3)	40	(9.5)		
Konkomba	16	(9.8)	33	(12.8)	49	(11.7)		
Others	7	(4.3)	17	(6.6)	24	(5.7)		
**School attended**							4.89	0.18
Baglo Ridge SHS	33	(20.2)	57	(22.2)	90	(21.4)		
Bueman SHS	51	(31.3)	57	(22.2)	108	(25.7)		
Father Dogli SHS	39	(23.9)	78	(30.4)	117	(27.9)		
Okadjakrom SHTS	40	(24.5)	65	(25.3)	105	(25.0)		
**Academic level**							2.49	0.114
SHS 2 (Second year)	104	(63.8)	144	(56.0)	248	(59.0)		
SHS 3 (Third year)	59	(36.2)	113	(44.0)	172	(41.0)		
**Course of study**							18.01	**0.012***
Business	8	(4.9)	5	(1.9)	13	(3.1)		
Fashion Design	5	(3.1)	22	(8.6)	27	(6.4)		
General Agriculture	2	(1.2)	6	(2.3)	8	(1.9)		
General Arts	64	(39.3)	84	(32.7)	148	(35.2)		
General Science	7	(4.3)	12	(4.7)	19	(4.5)		
Home Economics	14	(8.6)	46	(17.9)	60	(14.3)		
Technical	57	(35.0)	78	(30.4)	135	(32.1)		
Visual Art	6	(3.7)	4	(1.6)	10	(2.4)		
**Residency status**							0.45	0.504
Resident	139	(85.3)	225	(87.5)	364	(86.7)		
Non-resident	24	(14.7)	32	(12.5)	56	(13.3)		
**Health related variables**								

Variables	Intention to vaccinate against COVID-19: No = 163 (38.8%) n (%)	Yes = 257 (61.2%) n (%)	Total = 420 (100%) n (%)	Chi-square (x^2)	p-value
**History of COVID-19**				7.25	**0.007***
No	158 (96.9)	231 (89.9)	389 (92.6)		
Yes	5 (3.1)	26 (10.1)	31 (7.4)		
**family history of underlying conditions**				1.74	0.187
No	151 (92.6)	228 (88.7)	379 (90.2)		
Yes	12 (7.4)	29 (11.3)	41 (9.8)		
**Perception of good health**				0.01	0.916
No	43 (26.4)	69 (26.8)	112 (26.7)		
Yes	120 (73.6)	188 (73.2)	308 (73.3)		
**Adherence to COVID-19 protocol**					
**Face mask usage**				0.13	0.716
No	58 (35.6)	87 (33.9)	145 (34.5)		
Yes	105 (64.4)	170 (66.1)	275 (65.5)		
**Handwashing practices**				5.40	**0.02***
No	20 (12.3)	15 (5.8)	35 (8.3)		
Yes	143 (87.7)	242 (94.2)	385 (91.7)		
**Social distancing practices**				10.15	**0.001***
No	82 (50.3)	89 (34.6)	171 (40.7)		
Yes	81 (49.7)	168 (65.4)	249 (59.3)		
**Hand sanitiser use**				0.31	0.581
No	36 (22.1)	51 (19.8)	87 (20.7)		
Yes	127 (77.9)	206 (80.2)	333 (79.3)		

*p-value less than 0.05

Table 3: Univariate analyses between HBM and TPB constructs and the intention to get vaccinated against COVID-19

	Intention to vaccinate against COVID-19				
**Variables**	**No (n=163)**	**Yes (n=257)**	**t-test**	**p-value (two-tail)**	**Effect size (Cohen's d)**
**HBM**	**Mean (std. dev.)**	**Mean (std. dev.)**			
Perceive susceptibility	11.19 (4.30)	12.06 (4.37)	-1.99	**0.047***	-0.20
Perceive severity	13.80 (2.58)	13.68 (2.49)	0.46	0.645	0.05
Perceive benefit	9.72 (3.30)	10.85 (3.02)	-3.60	**0.0004***	-0.36
Perceive barrier	13.75 (4.41)	12.91 (4.39)	1.90	0.058	0.19
Self-efficacy	9.04 (2.63)	10.49 (3.00)	-5.05	**<0.0001***	-0.51
Cues to action	16.11 (4.58)	16.69 (4.71)	-1.25	0.212	-0.13
**TPB**					
Attitudes	18.21 (3.68)	20.17 (3.51)	-5.47	**<0.0001***	-0.55
Subjective norms	14.93 (4.68)	17.51 (4.91)	-5.35	**<0.0001***	-0.54
Perceive behavioral control	7.42 (2.08)	7.33 (2.11)	0.42	0.673	0.04

*p-value less than 0.05; std. dev = standard deviation

Table 4: Hierarchical logistic regression models predicting intention to vaccinate against COVID-19

Predictor Variables	Model 1: Health-related, Adherence and HBM		Model 2: Health-related, adherence and TPB		Model 3: Health-related, Adherence, HBM and TPB	
	AOR (95% CI)	p-value	AOR (95% CI)	p-value	AOR (95% CI)	p-value
**Block 1: Sociodemographic**						
Sex (Ref: Female)						
Male	0.78 (0.50 – 1.22)	0.275	0.72(0.46–1.13)	0.148	0.81(0.51 – 1.29)	0.371
Course (Ref: Academic)						
Technical/Applied	1.45(0.91–2.30)	0.12	1.71(1.07–2.73)	**0.025***	1.59(0.98 – 2.57)	0.061
Creative/Vocational	1.21(0.52–2.79)	0.661	1.38(0.59–3.18)	0.456	1.12(0.47 – 2.67)	0.804
**Block 2: Health- related**						
History of COVID-19 (Ref: No)						
Yes	3.55(1.25–10.12)	**0.018***	2.91(1.03–8.21)	**0.043***	3.26(1.11 – 9.56)	**0.032***
**Block 3: Adherence**						
Hand washing (Ref: No)						
Yes	1.51 (0.69 – 3.30)	0.299	1.70(0.79–3.63)	0.173	1.51(0.68 – 3.33)	0.313
Social distancing (Ref: No)						
Yes	1.94 (1.25 – 3.02)	**0.003***	1.73(1.11–2.69)	**0.015***	1.85(1.17 – 2.91)	**0.008***
**Block 4: HBM Constructs**						
Perceived susceptibility	1.03 (0.97 – 1.09)	0.342			1.01(0.95–1.06)	0.915
Perceived benefit	1.11(1.03–1.20)	**0.006***			1.04 (0.95–1.13)	0.384
Self-efficacy	1.21(1.12–1.30)	**<0.001***			1.20 (1.11-1.31)	**<0.001***
**Block 5: TPB Constructs**						
Attitude			1.13(1.06–1.20)	**<0.001***	1.10(1.03 – 1.18)	**0.004***
Subjective norms			1.08(1.03–1.13)	**0.001***	1.09(1.04 – 1.15)	**0.001***

*p-value less than 0.05; AOR = Adjusted odds ratio; CI = Confidence interval

Discussion

This study examined the prevalence and predictors of COVID-19 vaccine intention among SHS students in Jasikan Municipality, Ghana, using the HBM and TPB. The study employed a cross-sectional design, with data analysed using hierarchical logistic regression.

Based on the study population we assessed, more than three-fifth expressed an intention to vaccinate against COVID-19, while nearly two-fifth did not. This indicates a majority intention to vaccinate, but also a substantial proportion of vaccine hesitancy. This intention rate is higher than the 45.7% reluctance (and 54.3% intention) found in a broader national survey of Ghanaians conducted between March and April 2021 [41]. It is also higher than the 45% COVID-19 vaccine uptake observed among students at Takoradi Technical University, Ghana, where 55% had not received the vaccine [42]. Compared to other student populations globally, the 61.2% intention rate from our study is somewhat comparable to the 63.8% reported among college students in India [27] and the 57.8% willingness among Palestinian dental students in early 2021 [43]. However, it is lower than the average global pooled intention rate of 67.69% for COVID-19 vaccines [44] and the 73.2% average rate from a meta-analysis on TPB studies [35]. Significantly higher intentions were found among general population in Israel (80%), [37] households in Nigeria (>85%) [45], Italian university students (94.7%) [46] and Jakarta, Indonesia residents (93%) [34]. On the other hand, the intention rate is considerably higher than the 31.4% willingness reported in an Ethiopian study among the general population [47]. The 38.8% who did not express an intention to vaccinate in this study is also higher than the 33.2% average vaccine hesitancy rate reported in a systematic review of HBM studies [48]. The possible explanation to this observed intention rate could be that in the Jasikan Municipality,

where over half of the COVID-19 cases by December 2021 were among students [49], may have influenced their perceptions of risk, probably leading to a higher intention to vaccinate compared to some other populations. The study was also conducted among SHS students, an age group that might have different social dynamics and risk perceptions compared to older adults or general university students. Though most students intend to vaccinate, the substantial minority (38.8%) who do not, highlights the critical need for targeted public health strategies to address vaccine hesitancy within this specific demographic. Strategies should focus on understanding the underlying reasons for their reluctance.

Now, turning to the significant predictors identified, a history of COVID-19 infection was significantly associated with the intention to vaccinate. This finding aligns with a study in Jakarta, Indonesia, which showed that participants with family members previously diagnosed with COVID-19 were 1.5 times more likely to get vaccinated [34]. It also resonates with the finding that Palestinian dental students infected before positively influenced their attitude toward new vaccines [43]. However, it contrasts with a Bangladeshi study where the COVID-19 infection status of oneself and family members was not a significant predictor of vaccine hesitancy within the HBM framework [50]. A German study also found that individual risk perception did not significantly influence their intention to take up vaccination [51]. Experiencing COVID-19 firsthand, or seeing its impact on family, likely increases the perceived susceptibility of individuals to and severity of the disease [22]. This direct experience can make the threat of the virus more tangible and less abstract, thereby strengthening the motivation to seek protection through vaccination. Public health campaigns could incorporate testimonials or narratives from individuals who have recovered from COVID-19, emphasising their experience with the illness to highlight the continued

relevance of vaccination as a protective measure for those who may perceive the risk as low or abstract.

Being a student offering a technical or applied course was significant in Model 2, but lost significance in Model 3. The initial significance might be comparable to studies finding differences in vaccination intention based on the field of study, particularly medical or healthcare-related fields. For instance, studies found that being a medical student determines your decision to receive vaccination [46,51,52]. However, some studies have found no significant differences between students in healthcare and non-healthcare settings [53], or even higher acceptance in non-healthcare settings [27]. The disappearance of significance in Model 3 suggests that the effect of being in a technical or applied course might be mediated or explained away by the other psychological constructs when they are all considered together. For example, based on our study about more than half of the students who reported ever having contracted COVID-19 were enrolled in the Technical or Applied programmes. It is plausible that their own illness or witnessing the experiences of classmates who had the disease may have influenced their perceptions and beliefs about vaccination, contributing to the observed significance in Model 2. Once constructs such as perceived susceptibility, perceived severity, attitude and self-efficacy were included in Model 3, the explanatory power of course type may have been subsumed by these psychological variables.

Further, adherence to social distancing was also significantly associated with a higher intention to vaccinate. In the U.S. Northeast, clustering of vaccine-related attitudes and precautionary behaviours within social networks was reported [54]. That is, social network contacts following social distancing guidelines was significantly associated with the participant's intention to vaccinate [54]. This indicates that if the close social

contacts of an individual adhered to social distancing, it positively influenced that individual's intent to vaccinate. Individuals who regularly adhere to social distancing measures likely share an underlying predisposition towards health-protective behaviours and a higher degree of perceived risk or collective responsibility [51]. Their willingness to follow one public health guideline (social distancing) could translate into a similar willingness to adhere to another (vaccination) which suggests a consistent health-conscious mindset.

Among TPB constructs, attitude was a significant predictor of intention to vaccinate. This finding is strongly consistent with a systematic review and meta-analysis on the TPB, which identified attitude as having the strongest association with vaccination intention [35]. Other studies including one in Bangladesh [50], another among German university students [51] and Palestinian dental students [43] have linked positive attitude to vaccination intention. According to the TPB, the attitude of an individual towards a behaviour is a direct determinant of their intention to perform that behaviour [35–37]. A positive attitude reflects a belief that vaccination is desirable or beneficial [37]. Public health campaigns should strategically focus on shaping positive vaccination attitudes by emphasising the direct benefits of vaccination, such as protection from severe illness, hospitalisation, and death, and addressing common concerns about vaccine safety and efficacy. Public health campaigns should focus on shaping positive attitudes toward COVID-19 vaccination by reinforcing its perceived benefits such as protection from severe illness, and improved well-being among students. Health promotion activities should also directly address misinformation and misconceptions, including religious or apocalyptic beliefs and fears of long-term harm, while clarifying the safety, effectiveness, and ease of accessing the vaccine.

Subjective norms were a significant predictor of intention to vaccinate. A meta-analysis on TPB showed subjective norms had a strong effect size on vaccination intention [35]. Also, a Bangladeshi study confirmed that familial support (a component of subjective norms) significantly reduced vaccine hesitancy [50]. Furthermore, studies in the US highlighted that recommendations from family and friends were important mediators explaining demographic differences in vaccination uptake, and parental encouragement was a strong predictor of uptake among college students [55]. A national survey in Ghana also noted that encouragement by trusted community leaders increased vaccination intention [41]. Recommendations from family members and doctors as well significantly affect these intentions [56–58]. For students, social influence from peers and family can be particularly potent. If they perceive that their social circle or trusted figures approve of vaccination, they are more likely to intend to get vaccinated. Encouraging vaccinated individuals to share their positive experiences and involving respected community, religious, or educational leaders as "vaccine champions" can amplify the impact of subjective norms [49].

In this study, except for self-efficacy, none of the other HBM constructs (perceived susceptibility, perceived severity, perceived benefits, perceived barriers, cues to action) retained statistical significance in the final model (Model 3) which contrasts a systematic review and meta-analysis study [44]. Studies have consistently identified it as a positive predictor of vaccination intention [37,56]. High self-efficacy is crucial for adopting protective behaviours and can help individuals overcome perceived barriers. Some studies using single-item measures for self-efficacy reported a lesser impact [55]. Students who believe they can receive the COVID-19 vaccine on time, do not find it difficult to access vaccination services, and feel that taking other precautions may substitute for vaccination are more likely to express an intention to vaccinate.

These perceptions reflect their self-efficacy in managing the vaccination process. This confidence helps them overcome potential perceived barriers and translates directly into a stronger intention. Public health programs should focus on building self-efficacy by ensuring timely access to vaccination services, reducing difficulties in reaching vaccination centres, and clarifying that vaccination complements rather than replaces other preventive measures.

Grounding this study on both HBM and TPB was able to explain about a quarter (25.7%) of the variance (as indicated by Cragg & Uhler's R^2) which is considerably higher compared to using the HBM (19%) and TPB (19.8%) separately. Another study in Israel reported a significantly higher variance (78%) using same unified model [37]. The possible explanation could be linked to the timeframe of data collection. For instance, this study was conducted around the third quarter of 2022, where COVID-19 vaccines had been available for some time, and real-world experiences with the virus and vaccines would have been common. When you compare the Israeli situation, the study was conducted during the second quarter of 2020 which was at the point when the COVID-19 vaccines were still under development and not available publicly [37,59]. The study measured intention to receive a future vaccine, which might reflect a more theoretical or initial willingness based on perceived threat rather than practical considerations or actual experiences. When vaccinations are hypothetical, intentions of individuals might be more directly predicted by perceived susceptibility, severity, and benefits, as these are primary drivers in the early stages of a health threat [60]. Once vaccines become available, and people gain personal or indirect experience, other factors such as perceived side effects, actual accessibility, changing risk perceptions, and the evolving public narrative (including misinformation) can become more dominant and complex influences, possibly reducing the explanatory power of

initial behavioural models [61–63]. In support of this, the Israeli study found perceived benefits, perceived severity, and cues to action to be significant predictors at a time when the vaccine was not yet available [37] while these same constructs were not significant in this study.

Limitations

This study has limitations that need to be considered while interpreting the findings. The use of a cross-sectional design limits causal inference; the identified associations between predictors and intention to vaccinate cannot be interpreted as causal relationships. Additionally, all data obtained from participants were self-reported, which introduces the risk of social desirability and recall biases. The study assessed intention to vaccinate rather than actual vaccine uptake. Although intention is a known predictor of behaviour, it does not always translate into action. The study was conducted in a single municipality and involved only Senior High School students; therefore, the findings may not be generalisable to out-of-school students in other regions of Ghana. Even though several important predictors were assessed using the HBM and TPB frameworks, unmeasured factors such as parental influence, media exposure, or political trust may also influence vaccination intention and might have improved the proportion of explained variance if included. Furthermore, the internal consistency of some constructs in the behavioural models performed below the commonly accepted reliability threshold, which may have influenced the strength of observed associations. Finally, the study was conducted after vaccines had become available, and some students had already received at least one dose. While this reflects real-world exposure and enhances the practical relevance of the findings, it may have influenced intention responses, limiting interpretation of intention as a purely

prospective construct. Public narratives around COVID-19 had also evolved, and shifting risk perceptions or circulating misinformation during that period may have further shaped the responses of the participants.

In managing these limitations, the study employed a robust multistage sampling technique to enhance representativeness within the selected municipality. Although the questionnaires were self-administered, trained research assistants took time to explain the purpose of the study and clarify each item before students responded. This approach helped ensure consistency in understanding and reduced the risk of misinterpretation. Anonymity and confidentiality were emphasised to minimise social desirability bias. In addition, the use of two complementary behavioural models, HBM and TPB, allowed for a comprehensive assessment of key psychological constructs. The structured questionnaire contributed to improved data quality. Though the study was conducted in one municipality and measured intention rather than actual uptake, the methodological approach strengthens the relevance and potential applicability of the findings to similar settings.

Conclusions

Overall, about six in ten students expressed an intention to vaccinate against COVID-19. This intention was influenced by prior experience with COVID-19, adherence to social distancing measures, positive vaccination attitudes, perceived social expectations, and confidence in one's ability to get vaccinated. It is therefore recommended that public health campaigns should engage students with personal experience of COVID-19 to share their perspectives and encourage peer uptake. Health promotion strategies that target attitudes, social influences, and students'

confidence in their ability to get vaccinated should be intensified to improve vaccine acceptance.

List of abbreviations

AOR – Adjusted Odds Ratio; CI – Confidence Interval; HBM – Health Belief Model; PBC – Planned Behavioural Control; SHS – Senior High School; SHTS – Senior High Technical School; TPB – Theory of Planned Behaviour; TVET - Technical and Vocational Education Training; WHO – World Health Organisation.

Consent for publication

Not applicable.

Data availability

All data supporting the findings of this study are fully contained within the manuscript.

Competing interests

The authors have declared that no competing interests exist.

Funding

The author(s) received no specific funding for this work.

Authors' contributions

HA, MG, and VOC conceptualized the study. HA, MG, SAB, and VOC developed the methodology. HA, MG, and VOC conducted the investigation. HA, MG, and SAB curated the data. HA, MG, SAB, SAZ, and VOC performed the formal analysis. HA, MG, GSA, SAZ, JA, and VOC contributed to data visualization. HA and MG drafted the original manuscript. HA, MG, SAB, GSA, SAZ, JA, IA, and VOC reviewed and edited the manuscript. All authors read and approved the final manuscript.

Supplementary information

S1 Data. A minimal dataset necessary to support the findings presented in the manuscript.

S1 Table. Model fit statistics for hierarchical logistic regression models predicting intention to vaccinate against COVID-19

S2 Table. A summary of variable descriptions, value labels, and categorical classifications as used in the Stata analysis of the dataset supporting the findings presented in the manuscript.

Acknowledgements

This research was conducted as part of Hudatu Ahmed's Bachelor of Public Health (Nutrition) degree at the Fred N. Binka School of Public Health, University of Health and Allied Sciences, Ghana. The authors acknowledge the Education Director, headteachers, and teachers for their support in facilitating data collection. Appreciation is also extended to the trained research assistants and health staff for their role in data collection, and to all study participants for their participation.

References

1. United Nations International Children's Emergency Fund (UNICEF). Immunization [Internet]. 2022 [cited 2025 Jun 17]. Available from: https://www.unicef.org/immunization

2. WHO. 10 global health issues to track in 2021 [Internet]. 2020 [cited 2025 Jun 17]. Available from: https://www.who.int/news-room/spotlight/10-global-health-issues-to-track-in-2021

3. Public Health Emergency of International Concern (PHEIC). 2020.

4. WHO. WHO Coronavirus (COVID-19) Dashboard [Internet]. 2023 [cited 2025 Jun 17]. Available from: https://data.who.int/dashboards/covid19/cases

5. RSTMH Country Ambassador. The COVID-19 situation in Ghana [Internet]. 2021 [cited 2025 Jun 17]. Available from: https://www.rstmh.org/news-blog/news/the-covid-19-situation-in-ghana

6. Kenu E, Frimpong J, Koram K. Responding to the COVID-19 pandemic in Ghana. Ghana Med J. 2020 Jun 30;54(2):72–3.

7. WHO. COVID-19 vaccine doses shipped by the COVAX Facility head to Ghana, marking beginning of global rollout [Internet]. Joint News Release. 2021 [cited 2025 Jun 17]. Available from: https://www.who.int/news/item/24-02-2021-covid-19-vaccine-doses-shipped-by-the-covax-facility-head-to-ghana-marking-beginning-of-global-rollout

8. WHO Africa. Ghana's President receives first shot of historic COVAX vaccine to launch vaccination campaign [Internet]. 2021 [cited 2025 Jun 17]. Available

from: https://www.afro.who.int/news/ghanas-president-receives-first-shot-historic-covax-vaccine-launch-vaccination-campaign

9. WHO. WHO Coronavirus (COVID-19) Dashboard [Internet]. Vaccine. 2023 [cited 2025 Jun 17]. Available from: https://data.who.int/dashboards/covid19/vaccines

10. Ruggeri K, Vanderslott S, Yamada Y, Argyris YA, Većkalov B, Boggio PS, et al. Behavioural interventions to reduce vaccine hesitancy driven by misinformation on social media. BMJ. 2024 Jan 16;e076542.

11. Sweileh WM. Bibliometric analysis of global scientific literature on vaccine hesitancy in peer-reviewed journals (1990–2019). BMC Public Health. 2020 Dec 17;20(1):1252.

12. Wu Q, Dudley MZ, Chen X, Bai X, Dong K, Zhuang T, et al. Evaluation of the safety profile of COVID-19 vaccines: a rapid review. BMC Med. 2021 Dec 28;19(1):173.

13. Liu Q, Qin C, Liu M, Liu J. Effectiveness and safety of SARS-CoV-2 vaccine in real-world studies: a systematic review and meta-analysis. Infect Dis Poverty. 2021 Dec 14;10(1):132.

14. Kuatewo M, Ebelin W, Doegah PT, Aberese-Ako M, Lissah S, Kpordorlor AG, et al. Fake news, misinformation, vaccine hesitancy and the role of community engagement in COVID-19 vaccine acceptance in Southern Ghana. PLoS One. 2025 Jun 10;20(6):e0316969.

15. Garett R, Young SD. Online misinformation and vaccine hesitancy. Transl Behav Med. 2021 Dec 14;11(12):2194–9.

16. Wilson SL, Wiysonge C. Social media and vaccine hesitancy. BMJ Glob Health. 2020 Oct;5(10):e004206.

17. Hydarav R, Igweonu O, Anand S, Jemimah M, Merdekios A, Abrar L, et al. Establishment and Use of Polio Communication Network in Response to Polio in Outbreak Countries of the Horn of Africa: 2013–2014. J Immunol Sci. 2021 Apr 30;Special Issue(2):48–54.

18. Yahya M. Polio vaccines—“no thank you!” barriers to polio eradication in Northern Nigeria. Afr Aff (Lond). 2007 Apr 1;106(423):185–204.

19. Jegede AS. What Led to the Nigerian Boycott of the Polio Vaccination Campaign? PLoS Med. 2007 Mar 20;4(3):e73.

20. Roy DN, Biswas M, Islam E, Azam MdS. Potential factors influencing COVID-19 vaccine acceptance and hesitancy: A systematic review. PLoS One. 2022 Mar 23;17(3):e0265496.

21. Amo-Adjei J, Nurzhynska A, Essuman R, Lohiniva AL. Trust and willingness towards COVID-19 vaccine uptake: a mixed-method study in Ghana, 2021. Archives of Public Health. 2022 Feb 21;80(1):64.

22. Kumi GA, Gohoho M, Aku FY. Determinants of COVID-19 vaccine hesitancy among healthcare personnel in a secondary-level health facility in Ghana. PLOS Global Public Health [Internet]. 2025 Sep 1 [cited 2025 Oct 7];5(9):e0004980. Available from: https://journals.plos.org/globalpublichealth/article?id=10.1371/journal.pgph.0004980

23. Leal Filho W, Wall T, Rayman-Bacchus L, Mifsud M, Pritchard DJ, Lovren VO, et al. Impacts of COVID-19 and social isolation on academic staff and students at universities: a cross-sectional study. BMC Public Health. 2021 Dec 24;21(1):1213.

24. Dietz P, Reichel JL, Edelmann D, Werner AM, Tibubos AN, Schäfer M, et al. A Systematic Umbrella Review on the Epidemiology of Modifiable Health Influencing Factors and on Health Promoting Interventions Among University Students. Front Public Health. 2020 Apr 28;8.

25. Schäfer M, Stark B, Werner AM, Tibubos AN, Reichel JL, Pfirrmann D, et al. Health Information Seeking Among University Students Before and During the Corona Crisis—Findings From Germany. Front Public Health. 2021 Jan 25;8.

26. Ioannidis JPA. COVID-19 vaccination in children and university students. Eur J Clin Invest. 2021 Nov 26;51(11).

27. Jain L, Vij J, Satapathy P, Chakrapani V, Patro B, Kar SS, et al. Factors Influencing COVID-19 Vaccination Intentions Among College Students: A Cross-Sectional Study in India. Front Public Health. 2021 Dec 15;9.

28. Reuter PR, Forster BL, Kruger BJ. A longitudinal study of the impact of COVID-19 restrictions on students' health behavior, mental health and emotional well-being. PeerJ. 2021 Dec 14;9:e12528.

29. Fruehwirth JC, Biswas S, Perreira KM. The Covid-19 pandemic and mental health of first-year college students: Examining the effect of Covid-19 stressors using longitudinal data. PLoS One. 2021 Mar 5;16(3):e0247999.

30. Viner R, Russell S, Saulle R, Croker H, Stansfield C, Packer J, et al. School Closures During Social Lockdown and Mental Health, Health Behaviors, and Well-being Among Children and Adolescents During the First COVID-19 Wave. JAMA Pediatr. 2022 Apr 1;176(4):400.

31. Aucejo EM, French J, Ugalde Araya MP, Zafar B. The impact of COVID-19 on student experiences and expectations: Evidence from a survey. J Public Econ [Internet]. 2020 Nov 1 [cited 2025 Jun 17];191:104271. Available from: https://www.sciencedirect.com/science/article/pii/S0047272720301353

32. Zartaloudi A. Health Belief Model (HBM) and vaccination during pandemics. European Psychiatry. 2022 Jun 1;65(S1):S308–S308.

33. Limbu YB, Gautam RK. How Well the Constructs of Health Belief Model Predict Vaccination Intention: A Systematic Review on COVID-19 Primary Series and Booster Vaccines. Vaccines (Basel). 2023 Apr 7;11(4):816.

34. Hidayana I, Amir S, Pelupessy DC, Rahvenia Z. Using a health belief model to assess COVID-19 vaccine intention and hesitancy in Jakarta, Indonesia. PLOS Global Public Health. 2022 Oct 28;2(10):e0000934.

35. Limbu YB, Gautam RK, Zhou W. Predicting Vaccination Intention against COVID-19 Using Theory of Planned Behavior: A Systematic Review and Meta-Analysis. Vaccines (Basel). 2022 Nov 26;10(12):2026.

36. Wolff K. COVID-19 Vaccination Intentions: The Theory of Planned Behavior, Optimistic Bias, and Anticipated Regret. Front Psychol. 2021 Jun 16;12.

37. Shmueli L. Predicting intention to receive COVID-19 vaccine among the general population using the health belief model and the theory of planned behavior model. BMC Public Health. 2021 Dec 26;21(1):804.

38. Hajian-Tilaki K. Sample size estimation in epidemiologic studies. Caspian J Intern Med. 2011;2(4):289–98.

39. Acheampong T, Akorsikumah EA, Osae-Kwapong J, Khalid M, Appiah A, Amuasi JH. Examining Vaccine Hesitancy in Sub-Saharan Africa: A Survey of the Knowledge and Attitudes among Adults to Receive COVID-19 Vaccines in Ghana. Vaccines (Basel). 2021 Jul 22;9(8):814.

40. Bakker A, Cai J, English L, Kaiser G, Mesa V, Van Dooren W. Beyond small, medium, or large: points of consideration when interpreting effect sizes. Educational Studies in Mathematics [Internet]. 2019 Sep 15 [cited 2025 Oct 4];102(1):1–8. Available from: https://link.springer.com/article/10.1007/s10649-019-09908-4

41. Udor R, Eshun SN, Tampah-Naah AM. Factors Influencing Intentions and Acceptance of COVID-19 Vaccines in Ghana. Ghana Journal of Development Studies. 2023 Jun 1;20(1):252–66.

42. Lokko CNM, . LFO, Arhin R. COVID-19 vaccine uptake among students at Takoradi Technical University Ghana: prevalence and predictors. International Journal of General Medicine and Pharmacy (IJGMP) [Internet]. 2025 [cited 2025 Jun 22];14(1):27–36. Available from: http://www.iaset.us/index.php/download/archives/12-02-2025-1739345312-8-IJGMP-abstract-2.%20ABS%20IJGMP%20-%20COVID-19%20VACCINE%20UPTAKE%20AMONG%20STUDENTS%20AT%20TAKO

RADI%20TECHNICAL%20UNIVERSITY,%20GHANA%20PREVALENCE%20AND%20PREDICTORS.pdf

43. Kateeb E, Danadneh M, Pokorná A, Klugarová J, Abdulqader H, Klugar M, et al. Predictors of willingness to receive covid-19 vaccine: Cross-sectional study of palestinian dental students. Vaccines (Basel). 2021 Sep 1;9(9).

44. Yenew C, Dessie AM, Gebeyehu AA, Genet A. Intention to receive COVID-19 vaccine and its health belief model (HBM)-based predictors: A systematic review and meta-analysis. Hum Vaccin Immunother. 2023 Jan 2;19(1).

45. Eyawo O, Ugoji UC, Pan S, Oyibo P, Rehman A, Mahboob M, et al. Predictors of the willingness to accept a free COVID-19 vaccine among households in Nigeria. Vaccine. 2024 Oct;42(23):126225.

46. Pastorino R, Villani L, Mariani M, Ricciardi W, Graffigna G, Boccia S. Impact of COVID-19 Pandemic on Flu and COVID-19 Vaccination Intentions among University Students. Vaccines (Basel). 2021 Jan 20;9(2):70.

47. Belsti Y, Gela YY, Dagnew YAB, Getnet M, Seid MA, Diress M, et al. Willingness of ethiopian population to receive COVID-19 vaccine. J Multidiscip Healthc. 2021;14:1233–43.

48. Limbu YB, Gautam RK, Pham L. The Health Belief Model Applied to COVID-19 Vaccine Hesitancy: A Systematic Review. Vaccines (Basel). 2022 Jun 18;10(6):973.

49. Ghana Field Epidemiology and Laboratory Training Programme. Fifth Ghana FELTP Scientific Conference and 15th Anniversary Celebration Conference Proceedings. In: Journal of Interventional Epidemiology and Public Health

[Internet]. 2024. Available from: https://doi.org/10.37432/jieph.supp.2023.7.1.08.

50. Piya FL, Amin S, Das A, Kabir MA. Impacts of COVID-19 on the Education, Life and Mental Health of Students in Bangladesh. Int J Environ Res Public Health. 2022;19(2).

51. Schäfer M, Stark B, Werner AM, Mülder LM, Heller S, Reichel JL, et al. Determinants of university students' COVID-19 vaccination intentions and behavior. Sci Rep. 2022 Oct 27;12(1):18067.

52. Tavolacci MP, Dechelotte P, Ladner J. COVID-19 Vaccine Acceptance, Hesitancy, and Resistancy among University Students in France. Vaccines (Basel). 2021 Jun 15;9(6):654.

53. Barello S, Nania T, Dellafiore F, Graffigna G, Caruso R. 'Vaccine hesitancy' among university students in Italy during the COVID-19 pandemic. Eur J Epidemiol. 2020 Aug 6;35(8):781–3.

54. Khanna AS, Monnig MA, Clark SE, Monti PM. Social network characteristics of COVID-19 vaccination and preventive health behaviors: Cross-sectional findings from the US northeast during the early COVID-19 pandemic. Vaccine X. 2024 Dec;21:100577.

55. Huang Q, Abad N, Bonner KE, Baack B, Petrin R, Hendrich MA, et al. Explaining demographic differences in COVID-19 vaccination stage in the United States – April-May 2021. Prev Med (Baltim). 2023 Jan;166:107341.

56. Li Z, Sun X. Social factors influencing behavioral intentions to vaccinate: personality traits and cues to action. Front Psychol. 2025 Jan 23;16.

57. Gargano LM, Underwood NL, Sales JM, Seib K, Morfaw C, Murray D, et al. Influence of sources of information about influenza vaccine on parental attitudes and adolescent vaccine receipt. Hum Vaccin Immunother. 2015 Jul 3;11(7):1641–7.

58. Ellingson MK, Bednarczyk RA, O'Leary ST, Schwartz JL, Shapiro ED, Niccolai LM. Understanding the Factors Influencing Health Care Provider Recommendations about Adolescent Vaccines: A Proposed Framework. J Behav Med. 2023 Apr 22;46(1–2):356–65.

59. WHO. More than 150 countries engaged in COVID-19 vaccine global access facility [Internet]. News release. 2020 [cited 2025 Jun 22]. Available from: https://www.who.int/news/item/15-07-2020-more-than-150-countries-engaged-in-covid-19-vaccine-global-access-facility

60. Zampetakis LA, Melas C. The health belief model predicts vaccination intentions against COVID-19: A survey experiment approach. Appl Psychol Health Well Being. 2021 May 26;13(2):469–84.

61. Lee Y, Park K, Shin J, Oh J, Jang Y, You M. Factors Affecting the Public Intention to Repeat the COVID-19 Vaccination: Implications for Vaccine Communication. Healthcare. 2023 Apr 26;11(9):1235.

62. Borah P, Xiao X, Vishnevskaya A, Su Y. Narrative versus statistical messages: The interplay of perceived susceptibility and misperceptions on vaccine intention. Current Psychology. 2024 May 2;43(20):18674–89.

63. Kuru O, Stecula D, Lu H, Ophir Y, Chan M pui S, Winneg K, et al. The effects of scientific messages and narratives about vaccination. PLoS One. 2021 Mar 24;16(3):e0248328.

S1 Table

Click here to access/download
Supporting Information
S1 Table. Model fit statistics.docx

S2 Table

Click here to access/download
Supporting Information
S2 Table. Variable_dictionary.docx

S1 Data

Click here to access/download
Supporting Information
S1 Data.xls
