# Supplementary material for: Predictors of COVID-19 vaccination intention among students in Ghana: An application of the Health Belief Model and Theory of Planned Behaviour
Source: PLOS Glob Public Health. 2025 Dec 29;5(12):e0005561. doi: 10.1371/journal.pgph.0005561 (PMC12747339; doi:10.1371/journal.pgph.0005561)
Supplement: Attachment — Submitted filename: Response to Reviewers.docx [file pgph.0005561.s007.docx]

**Manuscript ID: PGPH-D-25-03045**

| **Comments** | **Responses** |
| --- | --- |
| We are grateful to the editor and reviewer for their comments, which have improved the quality and scientific rigor of our manuscript. We also appreciate the opportunity to revise and resubmit our work. | |
| **Journal Requirements:** |  |
| 1. Your manuscript is missing the following sections: Abstract. Please ensure these are present, and in the correct order, and that any references to subheadings in your main text are correct. An outline of the required sections can be consulted in our submission guidelines here: | The abstract section has now been added to the revised manuscript, structured according to PLOS Global Public Health requirements. All subsequent sections have been checked to ensure they follow the correct order and that references to subheadings are consistent with the journal’s submission guidelines. Please see **line 23.** |
| 2. Please provide separate figure files in .tif or .eps format. | All figures have been revised and attached as separate files to the submission. |
| **Title and Abstract** |  |
| Lines 1–15: Title is clear but overly long. Suggest simplifying to: • “Predictors of COVID-19 vaccination intention among students in Ghana: An application of the Health Belief Model and Theory of Planned Behaviour.” | The title has been revised as suggested. The new title reads: “*Predictors of COVID-19 vaccination intention among students in Ghana: An application of the Health Belief Model and Theory of Planned Behaviour*.”. Please see **lines 1-2**. |
| • Abstract (Lines 18–51): The background section repeats detailed context (e.g., “in 2021 most COVID-19 cases…”), which should be shortened. | The background section of the abstract has been revised to remove excessive contextual detail and improve conciseness. Please see **lines 24 – 30.** |
| • Methods lack clarity about how HBM and TPB constructs were operationalised—specify how scales were developed. | The Methods section has been revised to clarify the operationalisation of HBM and TPB constructs. Items measuring each construct were obtained through literature review and adapted for relevance to our context. Please see **lines 35-36**. |
| • Conclusion is appropriate but should explicitly link findings to potential interventions. | The conclusion has been revised to link findings to potential interventions. Please see **lines 49-51.** |
| Age mean and SD should be reported. | We have now reported the mean and standard deviation of participants’ age in the abstract. Please **see line 41.** |
| How many key words re allowed? | Keywords have been limited to 5. See **line 52.** |
| **Introduction (Lines 54–137)** |  |
| • Lines 54–61: The first paragraph reads as a general background on vaccines; it can be shortened to 3–4 sentences. | The first paragraph has been revised and shortened to improve focus and flow. It now highlights the public health importance of vaccinations and the dependence of their impact on public acceptance. See **lines 55-67.** |
| • Lines 62–91: Context on Ghana’s COVID-19 response is too detailed (dates, COVAX timeline, etc.). Retain only what informs local vaccine hesitancy. | The section on Ghana’s COVID-19 response has been condensed to remove excessive detail on dates and timelines. It now focuses on information directly relevant to local vaccine hesitancy, including public attitudes, misinformation, and contextual factors influencing vaccination decisions. See **lines 68-86.** |
| • Lines 92–137: The justification for using both HBM and TPB is good but poorly synthesized—needs a clear conceptual rationale for integrating the two frameworks rather than describing them separately. | Thank you for the comment. We have revised the justification to clearly articulate why integrating HBM and TPB strengthens our conceptual framework. The revision now explains that HBM addresses students' cognitive evaluations of health threats and vaccination benefits, while TPB captures their motivation and perceived ability to act within social contexts. Together, these frameworks provide a comprehensive understanding of both individual risk perceptions and social influences on vaccination behavior, which neither model addresses adequately alone. Please see **lines 91-104**. |
| • End the section with explicit research objectives or hypotheses (currently missing). | The research objectives have been stated at the end of the introduction section in **lines 104-107**. |
| • Consider adding a conceptual diagram early in the Introduction showing the link between HBM, TPB, and intention. | The conceptual diagram showing how HBM and TPB constructs relate to students’ intention to vaccinate has been moved up to the Introduction. See **lines 94-96**. |
| This should read mask | Thanks for the correction. The entire paragraph had been expunged, maintaining essential details. |
| **Methods (Lines 139–270)** |  |
| • Study design (Lines 162–171): Clearly state why a cross-sectional design was chosen and its limitations for causal inference. | Thank you for the comment. The study used a cross-sectional design because of its ability to quickly assess vaccination intention and multiple associated factors at a single point in time. We have also acknowledged that this design does not allow for causal inference. See **lines 134-136**. |
| How did you addressed the issue of consent among this age group. | For those aged 18 years and above, written informed consent was obtained. For those aged 15–17 years, parental or guardian consent was secured in addition to written assent from the participants themselves. See **lines 114-116.** |
| **• Sample size (Lines 172–185):** |  |
| • The formula is presented, but the expression is broken and not formatted properly. | The formula has now been reformatted for clarity. Please see **line 151**. |
| • Add information on response rate and handling of non-response. | Information on response rate and handling of non-response has been added. See **lines 157-159.** |
| **• Data collection (Lines 186–197):** |  |
| • Clarify whether data collectors were blinded to study hypotheses. | We appreciate the editor’s observation. In our study, data were collected using self-administered questionnaires completed directly by participants. As such, research assistants were not involved in recording responses. While the assistants were aware of the study objectives, their role was limited to distributing and collecting the questionnaires. Therefore, blinding was not applicable in this context. Please see **lines** **166-168.** |
| • Describe translation, pretesting process, and reliability assessment for all items. | Thank you for your comment. The questionnaire was adapted from existing literature and administered in English, as all participants could speak and understand the language. It was pretested with a small group of students outside the study sample to check for clarity and understanding. Minor changes were made based on their feedback. See **lines 168 – 176.** Reliability of the HBM and TPB items was assessed using Cronbach’s alpha, with values ranging from 0.63 to 0.86 based on the main study data. These findings have been presented in Table 1. |
| In this line, it should be stated whether is interviewer or self-administered questionnaire. | The sentence in **line 169** has been updated to read “The self-administered questionnaire….” |
| **• Variables (Lines 198–231):** |  |
| • Many constructs are listed but not referenced—cite validated scales or justify modifications. | The items used to measure the HBM and TPB constructs were adapted from previously validated instruments used in similar studies on vaccine acceptance and health behaviour which has been cited in the manuscript. Minor modifications were made to tailor the items to the COVID-19 vaccination context and the target population. See **lines 206-213**. |
| • Dichotomisation of intention may lead to information loss; discuss this decision. | Thank you for your comment. We acknowledge that dichotomising the 5-point Likert scale may result in some loss of information, which is a limitation. However, this approach was chosen to simplify the analysis and facilitate clearer comparisons between participants who expressed intention to vaccinate (responses 1–3) and those who did not (responses 4–5). It also enables straightforward comparison of factors associated with vaccination intention. This method has been used in previous similar studies. We have now included this rationale in the manuscript in **lines 182-185**. |
| **Statistical analysis (Lines 250–270):** |  |
| Explain criteria for including variables in multivariable models (e.g., p<0.2 from univariate?). | Thank you for your comment. Univariate analyses were first conducted for all independent variables to assess their individual association with COVID-19 vaccination intention. Only variables with p < 0.05 in the univariate analyses were subsequently included in the multivariable hierarchical logistic regression models. See **lines 237 – 238**. |
| State how multicollinearity was checked. | Thank you for your comment. Multicollinearity among independent variables was assessed using the variance inflation factor (VIF). See lines **243-248** for details. |
| Clarify whether model assumptions (e.g., goodness of fit) were tested. | Goodness of fit was assessed using log likelihood values, LR chi square tests with related p values, and pseudo R2 measures including Cragg Uhler and Nagelkerke values (see S2 Table for details). See **line 248-250**. |
| Add description of missing data handling. | Any field that appeared blank was cross-checked with the hardcopy questionnaire and completed to address missingness. See **line 224-225.** |
| **Results (Lines 271–379)** |  |
| • Tables: Well-structured but lengthy. Some could be moved to supplementary materials (e.g., full univariate table). | The full univariate table has been moved to supplementary materials (see S1 table). |
| • Lines 318–343: Hierarchical models are described clearly, but the rationale for three-step modeling should be briefly restated. | A brief rationale has been stated which reads *"Hierarchical binary logistic regression was conducted in three steps to examine the contributions of different sets of predictors."* See **lines 300-302.** |
| **• Statistical interpretation:** |  |
| • Avoid redundant p-values when AORs and CIs are reported. | We have removed the extra p-values where AORs and confidence intervals are already shown. See **lines 302-325**. |
| • Emphasise magnitude and direction of effects, not just significance. | The results in **lines 285-296** have been revised to highlight both the magnitude (Cohen’s d) and direction of effects for each variable, in addition to statistical significance. |
| • Figures (e.g., Fig. 2) should be properly labeled with titles and legends conforming to PLOS format. | Thank you. We have updated the figures to conform to PLOS format. |
| **Discussion (Lines 381–533)** |  |
| • Lines 381–414: The first paragraph is too long; break it into subtopics (comparison with other studies, interpretation of findings). | The paragraph has been restructured into shorter sections covering comparison with other studies and interpretation of findings. See **lines 337 – 354.** |
| • Lines 415–433: Interpretation of “history of COVID-19 infection” as a predictor is good but too descriptive—consider theoretical explanation via “perceived susceptibility” and “cue to action.” | The section has been revised to include theoretical explanations through perceived susceptibility and cue to action. See **lines** **357-360**. |
| • Lines 433–479: Several comparisons with foreign studies are excessive. Summarise key consistencies and differences in one paragraph. | The section has been revised by consolidating the multiple foreign study comparisons into one concise paragraph that highlights only the main consistencies and differences. **See lines 366-368.** |
| • Lines 480–504: The interpretation of self-efficacy and subjective norms is strong—expand on implications for intervention design. | Thank you for acknowledging the strength of our interpretation. This section has been revised by adding clear implications for intervention design, highlighting how subjective norms (in **lines 415-417**) and self-efficacy (in **lines 429-432)** can guide practical strategies to improve vaccination uptake. |
| • Lines 511–533: The discussion of model explanatory power (R² = 25.7%) is informative but needs statistical context—consider referencing similar behavioral studies in Africa. | The discussion has been revised to provide statistical context by comparing the explanatory power of our model (R² = 25.7%) with similar behavioral studies in Ghana, which reported variance explained between 35% and 62.5% among the general population, other adult populations, and healthcare workers. See **lines 435-437.** |
| • Limitations (Lines 535–569): Comprehensive but verbose. Condense to five concise sentences, prioritising: cross-sectional design, self-report bias, limited generalisability, and moderate internal reliability. | We appreciate the reviewer’s comments. The limitations of our study include its cross-sectional design, reliance on self-reported data, limited generalizability and moderate internal reliability. See **lines 446-452.** |
| Conclusion (Lines 571–579): Currently reiterates findings; should include specific actionable recommendations for policymakers or school-based vaccination programmes. For instance, “Future interventions should strengthen students’ self-efficacy and leverage social influence through peer and teacher engagement to enhance vaccine uptake in educational settings.” | Thank you for the comment. The conclusion has been revised to include specific actionable recommendations for policymakers and school-based vaccination programmes in **lines 457-461**. |
| **Technical and Formatting Issues** |  |
| • Referencing: Inconsistent citation style; some references appear as numeric, others as author–year in-text (e.g., [35,36]). Conform to PLOS Vancouver format. | All in-text citations and reference list have been revised to conform consistently to the PLOS Vancouver style. |
| • Language and grammar: Frequent redundancy, long sentences, and inconsistent tense usage. Recommend full professional language editing. | The manuscript has undergone thorough language and grammar editing to remove redundancies, shorten long sentences, and ensure consistent tense usage throughout. |
| • Tables/Figures: Ensure all are cited in order and formatted per journal templates. | All tables and figures have been checked to ensure they are cited sequentially in the text and formatted according to the journal’s template requirements. |
| • Data availability: Statement is acceptable but should include a repository link (e.g., OSF, Dryad) as per PLOS policy. | Thank you for the comment. The data availability statement has been updated to indicate that all anonymised data analysed in this study are included as Supporting Information (S1 Data) in **lines 465-467**. |
